# Cancer Patients' Self-Reported Attitudes About the Internet

**DOI:** 10.2196/jmir.7.3.e22

**Published:** 2005-07-01

**Authors:** Sheryl P LaCoursiere, M Tish Knobf, Ruth McCorkle

**Affiliations:** ^1^Center for Excellence in Chronic Illness CareYale University School of NursingYale UniversityNew Haven, CTUSA

**Keywords:** Breast neoplasms, Internet, information dissemination, computer communication networks, trust, disclosure, social support, Health Insurance Portability and Accountability Act

## Abstract

**Background:**

Increasing numbers of cancer patients are using the Internet, but little is known about their attitudes toward online health care.

**Objective:**

The purpose of this substudy was to analyze cancer patients' attitudes toward online health care.

**Methods:**

This was a substudy of 41 persons with cancer who used the Internet for health care information and support and who completed the Attitudes Toward Online Health Care (ATOHC) survey.

**Results:**

The majority of study participants were married, held graduate degrees, and had high incomes. Using a five-point Likert scale, means for the five dimensions of the ATOHC survey were as follows: community and news 3.22 (SD = 1.01), outcomes 3.20 (SD = 1.08), trusted information and advice 2.73 (SD = 0.66), self-efficacy in evaluating information and intention 3.46 (SD = 0.65), and disclosure 3.15 (SD = 1.06). The average response fell between “About half the time” and “Usually.” Favorite websites for content were Medscape and WebMD, while favorite sites for support were WebMD and Mediconsult.

**Conclusions:**

Respondents were generally eager to obtain and offer cancer information and support online, but they were skeptical of unknown sources. They were comfortable both giving and receiving information and support. Respondents were interested in the experiences of other patients and benefited by their direct and indirect interactions with them. Respondents felt that they coped better with their illness and experienced less uncertainty and anxiety as a result of their online experiences. They reported a certain level of trust, primarily for established reputable sources of information, and they were confident in their ability to evaluate the information, including research reports. In addition, cancer patients displayed a healthy skepticism when presented with the option of divulging their personal health information; however, they were willing to provide personal details if, as a result, a website provided them with individualized information.

## Introduction

The Internet is rapidly becoming an indispensable resource for persons with cancer. Over 50% of adults in the United States have searched online for health information, and 80% of all US Internet users have searched online for at least one major health topic. This makes searching for health information the third most common online activity after email and researching a product or service [1]. In the United States, there will be an estimated 1.3 million cases of cancer diagnosed in 2005; over 800000 persons will survive [2]. Considering the growing number of cancer survivors, which has increased from 3 million in 1971 (1.5% of the US population) to 9.8 million (3.5% of the US population) in 2001 [3], it is obvious that the need for information and support in cancer care is continually growing. Many persons newly diagnosed with cancer, as well as survivors, are turning to the Internet for assistance with their physical symptoms and psychological distress [4]. There is also a need for information and support for friends and family members, particularly caregivers [5].

Although the number of persons who seek cancer health care information online is difficult to measure [6], it has been reported as high as 41.5% of study participants [7]. Cancer patients who use the Internet are generally younger and have completed more education [8-10]. The type of cancer among Internet users varies. In one survey, 15% of respondents had digestive/gastrointestinal/bowel cancer, 11.7% had breast cancer, 11.3% had skin cancer, and 10.5% had genitourinary cancer [11]. Information and support needs change over time. In women with breast cancer, 49% reported using the Internet for information and support up to eight months after diagnosis, and 40% used it for up to 16 months [12]. Persons with cancer who seek online information and support add an average of 10 minutes to each clinical encounter with their oncologist. They may be more emotionally distressed as the information they uncover may cause them confusion and anxiety in addition to increasing their knowledge and sense of hope [13].

The Online Social Support Theory [14] and the Expanded Model of Health Care Consumer-Provider Interaction [15] suggest a relationship between online patient support groups and health outcomes. A specific framework for cancer patients has also been proposed [5]. Models such as these address the influential factors involved in seeking online health care, but they also address the complexity of factors that influence patients' attitudes about seeking online information and support in addition to traditional means. Unique factors to seeking health information online play an important role in a patient's level of involvement in online health venues. These factors include participation in a community with similar patients, the ability to rapidly obtain news and research findings, confidence in the ability to use the Internet, and the desire to gain some control of the illness through knowledge and support. This involvement with online health care is tempered by the decisions patients must make regarding their level of trust in this alternative system and their level of disclosure in order to obtain individualized information.

### Dimensions of Online Health Care

The concept of online health care encompasses a number of factors. Five particular factors that have been represented in the literature are community and news, outcomes, trusted information and advice, self-efficacy in evaluating information and intention, and disclosure.

#### Community and News

The Internet, as a collective entity of health professionals, peers, and other concerned international citizens, has responded to cancer patients' needs with a profusion of online community and news mechanisms for support and information. For psychosocial connection with others in a similar situation, cancer patients may choose from a variety of formats and venues: they can join email newsgroups and web-based discussion boards, or they can chat in real time. They can find general support groups or ones that are specific to their type or stage of cancer. Groups may be run by fellow patients, or, less frequently, by licensed health care providers. In addition to online community support, Internet news formats for cancer patients are steadily evolving and taking various forms, such as electronic newsletters and dedicated information sites, or a combination of community and news. For breast cancer alone, it is estimated that 2.4 million Web pages of information are available [16]. What features are rated most highly in a cancer website? In a survey of most preferred Web pages for prostate cancer patients, 59% cited websites that involved understanding diagnosis and treatment, 49% cited online help lines, and 44% preferred news sections [17]. In a survey of breast cancer sites rated by popularity in the search engine Google, 48% of the most popular sites offered opportunities for psychosocial support, 27% provided information on ongoing clinical trials, and 12% presented results of clinical trials [18]. Within Internet-based message boards, a frequent theme is concern regarding treatment, support, and side effects over time [19].

#### Outcomes

The ultimate test of the effectiveness of online health sites for cancer patients, particularly the effect of online support interventions, is their influence on health outcomes. Analysis of outcomes has been hampered by study designs that fail to distinguish between different types of support, for instance, support provided by peers, support provided with or without the presence of health care providers [20]. In a review of research literature related to online cancer support groups, Klemm et al [21] concluded that, in 9 out of 10 studies, persons with cancer coped better with their disease as a result of online participation. In general, persons with cancer enter online support groups significantly more depressed than their counterparts in face-to-face support groups [22]. In breast cancer patients, online support groups have been found to reduce depression and cancer-related trauma [23,24], loneliness [25], and reaction to pain [23]. They have also been associated with an increase in post-traumatic personal growth [23] and interpersonal social support [25].

#### Trusted Information and Advice

A very salient aspect of life for cancer patients is trust. Because of existential concerns and their need for hope, cancer patients are a vulnerable population [26,27]. Alternative treatments are often explored and may be considered an option to alleviate distressing physical and psychological symptoms [28]. This may precipitate a search for online information and support [26]. In one study, 63% of breast cancer patients researched alternative treatments, yet 53% were undecided about the trustworthiness of the information [10]. Trust of online sources among cancer patients may be influenced by age, time since diagnosis, ability to cope with having cancer, and the perceived credibility of the source [26]. In one study of breast cancer websites, only 31.6% offered information on the credentials of the site's operator [29].

#### Self-Efficacy in Evaluating Information and Intention

The knowledge gained from accessing online information and support and from participating in community and news venues of health websites can enhance one's self-efficacy and sense of empowerment. Through online health settings, cancer patients can develop a “social fitness” [30] as well as “cyber-agency” [31] concerning their disease that enables them to communicate more knowledgeably with health care providers. According to one study, 80% of cancer patients are interested in information related to treatment, 70% in conversations with physicians via the Internet, and 65% in online support groups [32]. Enhanced self-efficacy and a greater sense of control have the potential to increase patients' participation in their care, which may impact health outcomes.

#### Disclosure

In order to receive optimal benefit from online health venues, cancer patients may be asked to disclose personal health information, such as the stage of their cancer or the presence of metastasis. Because of the Healthcare Information Portability and Accountability Act (HIPAA) in the United States [33], disclosure of personally identifiable health information is tightly regulated in regard to research situations and existing health care organizations and agencies. Although websites that provide health care information may not be subject to this act in the strict sense of the law, it can be inferred that the passing of this legislation has raised consumer awareness. Even if organizations are not governed by law, ethical issues may still arise. Online users may encounter situations in which they will be unable to obtain the information they are seeking unless they disclose personal information about their health. This is particularly true in sites that present individualized information.

## Methods

### ATOHC Instrument

The Attitudes Toward Online Health Care (ATOHC) survey was developed to measure the attitudes of people who engage in online health care activities. The instrument was originally comprised of 51 items on a 5-point Likert scale. Possible responses included the following: 1 (Never), 2 (Seldom), 3 (About half the time), 4 (Usually), and 5 (Always) [34,35].

An exploratory factor analysis of 265 respondents who used online health care services was conducted using methodology outlined by Gable and Wolf [36]. According to RK Gable (March 2000), although 6 to 10 respondents are recommended per item, convergence occurred at 5.3. Five dimensions emerged: (1) community and news—supportive exchanges from other patients with similar conditions, and receipt of relevant information from other patients as well as health care professionals; (2) outcomes—psychological and physical changes in the individual as a result of having participated in online health care; (3) trusted information and advice—confidence in information provided by health authority figures and organizations; (4) self-efficacy in evaluating information and intention—individuals' belief in their ability to evaluate the quality of the information they receive, the qualifications of those providing it, and the intent of the requestor; and (5) disclosure—willingness to provide personally identifiable information. Alpha internal consistency reliability scores for the five dimensions were .95, .93, .84, .62, and .77, respectively [34]. Based on the results of poorly performing items in the factor analysis, the instrument was shortened to 42 items reflecting the five factors, for a final of 6.3 respondents per item. Only the 42 questions that were retained in the instrument were analyzed in this substudy; however, responses to one item of the instrument (“I trust online advice given by a Registered Pharmacist.”) were omitted due to a coding translation error from the Web page to the server. Therefore, only 41 items were analyzed, and possible scores for the ATOHC scale ranged from 41 to 205.

### Study Design

This was a descriptive study using a subsample of cancer patients from the total sample of those 265 persons who participated in the Attitudes Toward Online Health Care (ATOHC) study [34,35]. Two of the surveys were submitted twice on the website, leaving 263 usable questionnaires. Surveys in which participants listed a primary or secondary diagnosis of cancer were included in this substudy. There were a total of 41 surveys that met the criteria, with 39 persons listing a primary diagnosis of cancer, and five with a secondary diagnosis. Three persons listed both a primary and secondary diagnosis of cancer. A total of 39 persons with a primary diagnosis of cancer, and two with a secondary diagnosis of cancer are profiled.

Participants were recruited by one of three methods: (1) email discussion groups, (2) Web-based discussion groups, and (3) referrals from other websites. For the email discussion groups, a general invitation to participate was sent to a various groups asking for volunteers to complete the survey. An attempt was made to approach groups dealing with diverse medical conditions, such as heart disease, cancer, lupus, and those with general disability issues. In addition, messages were sent to a number of health professional discussion lists, including those for nurses, physicians, and physician assistants, asking those who personally utilized online health care services to volunteer. A similar procedure was followed for the Web-based discussion groups, with the exceptions that the message was posted on existing websites and potential respondents did not receive the notice automatically as they would with an email. For the referrals from other websites, arrangements were made with webmasters at two Internet health sites, Healthanswers.com and Askphysicians.com, to refer participants via links on these sites.

Data were collected from March 14, 2000 through March 28, 2000. Participants completed a demographic form and the ATOHC survey in a Web-based format. An additional free-text area asked the question, “What changes has receiving online health care information and support caused in your life?” Data were analyzed with the Statistical Package for the Social Sciences (SPSS), Version 11.0.0 [37]. Demographic characteristics, diagnoses, and favorite websites for content and support were analyzed by frequency tabulation. Means, standard deviations, and total scores overall and for each dimension were calculated from responses on the ATOHC scale for individual items.

## Results

### Demographic Characteristics

The mean age of respondents was 57.68 years (SD = 10.15; range 37–79). Slightly more than half, 53.7%, were male (n = 22), 78.9% were married (n = 32), and 90.2% were living in the United States (n = 37). Table 1 provides a summary of the demographic characteristics of the respondents.

**Table 1:** Demographic characteristics of respondents (N = 41)

	**n**	**%**
**Sex**		
Female	19	46.3
Male	22	53.7
**Marital Status**		
Married	33	80.5
Divorced	3	7.3
Single	3	7.3
Unspecified	2	2.9
**Education**		
High school diploma	5	12.2
Some college/associate's degree	11	26.9
Bachelor's degree	6	14.6
Graduate degree	18	43.9
Unspecified	1	2.4
**Annual Income**		
$5000–14999	3	7.3
$15000–34999	7	17.0
$35000–49999	7	17.1
$50000–74999	10	24.4
≥ $75000	7	17.1
Unspecified	7	17.1
**Work Status**		
Working full time	15	36.6
Not working: retired	13	31.7
Working part time (39 hours or less per week)	7	17.1
Not working: disabled or other reason	6	14.6
**Country**		
United States	37	90.2
Other	4	9.8

### Diagnoses

Participants were asked to select their primary and secondary diagnoses from a list by checking the relevant boxes on the online form. A separate area was provided to enter their diagnosis if it was not included in the list. For primary diagnoses, the majority of respondents elaborated on their type of cancer, whereas for secondary diagnoses, more non-cancer conditions were listed (Table 2).

**Table 2:** Respondents' listing of diagnoses and comorbid conditions (N = 41)

	**n**	**%**
**Primary Diagnosis**		
Cancer^*^	39	95.1
Cardiac	1	2.4
Fibromyalgia/chronic fatigue	1	2.4
**Secondary Diagnoses**		
Cancer^†^	5	12.2
Depression	3	7.3
Diabetes	2	4.9
Epstein-Barr	1	2.4
Huntington's Disease	1	2.4
Post-Traumatic Stress Disorder	1	2.4
Rhabdomyosarcoma	1	2.4
Seizure Disorder	1	2.4
Unspecified	26	63.4

^*^ Of the 39 persons listing cancer as a primary diagnosis, 11 specified the site (7 prostate, 2 breast, 1 colon, 1 chronic lymphocytic leukemia).

^†^ Of the 5 persons listing cancer as a secondary diagnosis, 2 specified the site (1 prostate, 1 kidney). Three respondents listed cancer as both a primary and secondary diagnosis. For the other two persons, the corresponding primary diagnoses were 1 Grave's disease, 1 unspecified.

### ATOHC Scale

In this sample, scores ranged from 50 to 172, with a mean of 128.46 (SD = 25.98). Thirty-six items had a scale range of 1–4, and 5 items had a range of 1–5. Pearson *r* correlations were performed between the continuous demographic variable, age, and total scores and factor scores. None were significant. The data are summarized in Table 3.

**Table 3:** ATOHC scores by dimension

**Dimension**	MeanScore^*^	**SD**	**ActualRange**	**PotentialRange**
Community and news	41.84	13.19	13–64	13–65
Outcomes	28.80	9.72	9–42	9–45
Trusted information and advice	27.27	6.58	10–37	10–50
Self-efficacy in evaluating information and intention	24.24	4.54	7–31	7–35
Disclosure	6.29	2.12	2–10	2–10

^*^ Higher scores indicate a greater degree of positive agreement.

The three highest ranked items were “I want to know how my online health information will be used before providing information” (mean = 3.93; SD = 1.29); “I am comfortable in evaluating the quality of online medical research reports” (mean = 3.83; SD = 0.89); and “I like to give online support to other patients who have my condition” (mean = 3.56; SD = 1.32). The item ranked lowest was “I tend to trust the products that other patients sell online” (mean = 1.63; SD = 0.77), followed by “I trust online summaries of health research articles even when I am not told who wrote them” (mean = 2.37; SD = 1.07). For the third lowest mean ranking, two items had a mean of 2.44: “I trust online healthcare advertising that has been sponsored by pharmaceutical companies” (SD = 0.95), and “I tend to trust a site more that has a seal of approval, even if I don't know the organization that is awarding it” (SD = 1.05). Outcomes related to depression fell between the highest and lowest scores. Table 4 presents the highly ranked items for each dimension, as well as the strength of the factor loadings of each item from the parent study.

**Table 4:** Highest mean scores on each dimension of the ATOHC survey (N = 41)

**Item** **No.**	**Item**	**Loading**	**Mean**	**SD**
**Factor I: Community and News (13 items)**			3.22	1.01
42	I like to give online support to other patients who have my condition.	.78	3.56	1.32
10	I like to participate in e-mail based discussion about my condition.	.75	3.54	1.25
14	I like to read online biographies of other patients that have had my condition.	.53	3.49	1.10
**Factor II: Outcomes (9 items)**			3.20	1.08
20	As a result of visiting health-related web sites, I have less uncertainty about my condition.	.69	3.51	1.21
40	As a result of visiting health-related web sites, I am better able to cope with my condition.	.80	3.46	1.16
12	As a result of visiting health-related web sites, I am less anxious about my condition.	.75	3.37	1.26
**Factor III: Trusted Information and Advice (10 items)**			2.73	0.66
49	I trust online reports of medical studies that have already been published in a journal.	.44	3.44	0.87
13	I trust online advice given by a Medical Doctor (MD).	.33	3.44	0.87
35	I trust a site that has been endorsed by a health authority.	.54	3.12	1.08
**Factor IV: Self-Efficacy in Evaluating Information and Intention (7 items)**			3.46	0.65
43	I want to know how my online health information will be used before providing information.	.36	3.93	1.29
11	I am comfortable in evaluating the quality of online medical research reports.	.48	3.83	0.89
41	I feel that online health information is at a comfortable comprehension level.	.36	3.61	0.89
**Factor V: Disclosure (2 items)**			3.15	1.06
3	I will disclose my email address to an online healthcare website.	.58	3.51	1.05
19	I will give my name to an online healthcare website if I will receive personalized information.	.44	2.78	1.31
**Entire scale**			3.13	0.63

### Favorite Websites for Content and Support

Participants were asked to select their favorite websites for content, as well as for support, from a drop-down menu offering a listing of popular sites. One of the options was “other,” in which case they could enter the name of a site using a text box. Medscape and WebMD were the most frequently mentioned favorite sites for content (31.7% each), while WebMD was the favorite site for support (17.1%). Favorite websites for content were primarily those sponsored by large organizations and by government agencies, such as the American Cancer Society and National Cancer Institute. Other favorite sites for support included a number of smaller, more specific sites such as Avon Crusade message boards and psa-rising.com. Favorite content and support sites are listed in Table 5.

**Table 5:** Favorite websites for content and support**

**Content**				**Support**			
		**n**	**%**			**n**	**%**
Medscape.com		13	31.7	WebMD.com		7	17.1
WebMD.com		13	31.7	Mediconsult.com		3	7.3
Intelihealth.com		2	4.9	Medscape.com		2	4.9
Mayohealth.com		2	4.9	Onhealth.com		2	4.9
Other		22^*^	51.2	Other		24^†^	53.7
	Prostate Help Mailing List (PHML)^‡^	3	7.2		PHML^‡^	3	7.3
	CancerLit (cancer.gov/search/cancer_literature/)	2	4.8		“Mailing lists”	2	4.8
	Cooleyville.com	2	4.8		MSN communities (groups.msn.com)	2	4.8
	Prostatepointers.org	2	4.8				
Sites listed once: about.com, Association of Cancer Online Resources (acor.org), American Cancer Society (cancer.org), drkoop.com, healthcentral.com, helioshealth.com, mediconsult.com, ostomyinternational.org, National Cancer Institute (nci.nih.gov), “Oncology Journals,” oncology.com, onhealth.com, prostate-cancer.org, “web2.airmail.net/lorac1”				Sites listed once: about.com, Association of Online Cancer Resources (acor.org), Avon Crusade Message Boards (avoncompany.com/women/avoncrusade/bbsindex.htm), cooleyville.com, “Doctors Guide to the Internet,” drkoop.com, “Heart Bypass and Transplant Support Board,” intelihealth.com, ostomyinternational.org, ivillage.com, Mass General NeuroWebForum (brain.hastypastry.net/forums/), “MS Breast Cancer Link,” onhealth.com, Patient to Physician (P2P) Mailing List^§^, Prostate Problems Mailing List (PPML)^‡^, prostate-cancer.org, prostatepointers.org, psa-rising.com, The Circle Mailing List^§^			

^**^ Websites listed in quotes are entries as listed by respondents that were not specific enough to identify a particular site or organization.

^*^ 21 persons listed “Other” favorite content sites. One respondent listed two sites; thus, there are 22 sites listed. Sites are in formats as listed by respondents.

^†^ 22 persons listed “Other” favorite support sites. Two respondents listed two sites each; thus, there are 24 sites listed. Sites are in formats as listed by respondents.

^‡^ The Prostate Help Mailing List (PHML) and Prostate Problems Mailing List (PPML) are sponsored by the Association of Cancer Online Resources (acor.org).

^§^ The Patient to Physician (P2P) Mailing List and The Circle Mailing List are sponsored by Prostatepointers.org.

## Discussion

The results of this analysis indicated that although respondents were generally married, they were otherwise from diverse backgrounds, with a tendency toward a higher level of education and income. This was consistent with previous studies. Based on the type of cancer and favorite websites, prostate cancer appeared to be the most common in this group, followed by breast cancer. Respondents utilized a variety of methods to obtain information and support about their cancer, including general medical sites such as WebMD, cancer-specific organizations such as the Association of Cancer Online Resources, patient-run cancer sites such Cooleyville.com, and specific mailing lists such as Prostate Problems Mailing List (PPML).

When responses to the ATOHC scale were analyzed, means for the five dimensions were more consistent than the means for individual items, ranging from 2.73 to 3.46 with standard deviations of 0.65 to 1.08. Mean scores for individual items demonstrated some variability, ranging from 1.63 to 3.93. However, the overall mean of 3.13 indicates that the average response was nearer to “About half the time” than “Usually” on the Likert scale. This demonstrates that respondents perceived some benefit as a result of obtaining health information and support online. This finding, in consonance with previous studies, is more reflective of the use of online health care as an adjunctive rather than a predominant modality of care. Although the use of online health care is rapidly increasing, obtaining health care information and support face-to-face remains the norm in the United States as well as internationally. Although the number of persons in the United States who have sought online health care information has just passed 51% of all adults, or 111 million people, a third of them accessed health information only on an infrequent basis, and not within the previous month [38].

There are several implications of this study for the care of cancer patients. First, patients are comfortable giving as well as receiving cancer information and support online and are comfortable evaluating it. They are interested in the experiences of other patients and derive benefit by interacting with them directly, through venues such as discussion boards and email lists, or indirectly, through activities such as reading biographies. Second, cancer patients perceived better outcomes after using online health information and support. This was manifested as being able to cope better with their condition, as well as having less uncertainty, anxiety, and, to a lesser extent, depression. Cancer patients have a certain level of trust in online information, primarily for information obtained from established reputable sources such as studies in journals and advice given by medical doctors. They also trust websites endorsed by health authorities. They are confident in their ability to evaluate information, including comprehension of research reports. In addition, cancer patients display a healthy skepticism when presented with the option of divulging personal health information. Some patients are willing to provide email addresses, and, if they receive personalized information, they are comfortable disclosing their identity.

Although results of the ATOHC survey with cancer patients are consistent with the parent survey, the current study needs to be replicated with a larger sample, and websites need to be validated to reflect their current Internet usage in light of mergers and acquisitions since the original study. In addition, correlation with variables such as coping and avoidance, as well as involvement in treatment decision-making [39], could shed light on the clinical outcomes of cancer patients who use the Internet compared to those who do not. Although cancer patients' attitudes about online health information are similar to those of persons with other chronic diseases, a comparison with other diagnoses may reveal unique characteristics and needs of cancer patients and assist in the development of evidence-based interventions. In addition, identification of what constitutes a successful outcome for differing populations of Internet users (eg, typical higher-income, more-educated users compared to users from an underserved population) would add to the growing knowledge base of persons with cancer who use the Internet. Based on the findings of this study, the results clearly demonstrate an untapped opportunity to improve the online information and support delivered to cancer patients. There are numerous opportunities along the treatment continuum to educate patients and family members about diagnostic and therapeutic options, as well as to correct misconceptions about cancer treatment. Although there has been significant progress in the provision of cancer treatment information and support, patients' needs often continue after the completion of primary therapy as they may have persistent symptoms, develop late effects, or face psychological challenges as they transition to survivorship. A percentage of patients also experience cancer recurrence. Any or all of these situations may prompt a need for additional information and support for patients and caregivers. There are also implications for HIPAA and the burgeoning use of web-based modalities for contact with health care providers [5]. Organizations and providers that deliver Internet-based care to cancer patients must be mindful of regulations related to disclosure and of distinctions that must be addressed in an electronic environment.

### Limitations

There were several limitations of this study which prevent the generalization of study results beyond the study sample population. First, the sample size is small and may not be representative of all persons with cancer who use the Internet. Second, the original ATOHC survey sample was comprised of a volunteer population of self-selected persons with chronic health issues. Third, because the cancer patients in the current study were primarily educated with higher incomes, generalization to underserved populations or to those who do not have Internet access cannot be made. Finally, since the data were collected in the year 2000, a number of the websites mentioned have consolidated, merged, or are no longer active. Thus, the study provides a snapshot of a point in time and cannot be inferred to be representative of current attitudes of cancer patients.

## Multimedia Appendix

The Attitudes Toward Online Health Care (ATOHC) Scale.
