# Supplementary material for: Cancer Patients' Self-Reported Attitudes About the Internet
Source: J Med Internet Res. 2005 Jul 1;7(3):e22. doi: 10.2196/jmir.7.3.e22 (PMC1550663; doi:10.2196/jmir.7.3.e22)
Supplement: Supplementary file 1 [file jmir_v7i3e22_app1.doc]

# Attitudes Toward Online Health Care (ATOHC) Scale

**Instructions**: Click on the circle in the area that most matches your attitude for each statement regarding online health care.

|  |  | **1 Never** | **2 Seldom** | **3 About half the time** | **4 Usually** | **5 Always** |
| --- | --- | --- | --- | --- | --- | --- |
| 1. | I like to participate in online chats with other patients that have my condition. | 1 | 2 | 3 | 4 | 5 |
| 2. | I will disclose my email address to an online healthcare website. | 1 | 2 | 3 | 4 | 5 |
| 3. | As a result of visiting health-related web sites, my physical health has improved because of advice I received online from another patient. | 1 | 2 | 3 | 4 | 5 |
| 4. | I like to participate on web-based message boards about my condition. | 1 | 2 | 3 | 4 | 5 |
| 5. | I read the privacy statements on healthcare websites. | 1 | 2 | 3 | 4 | 5 |
| 6. | I like to read health news about conditions other than mine. | 1 | 2 | 3 | 4 | 5 |
| 7. | I like to participate in e-mail based discussion about my condition. | 1 | 2 | 3 | 4 | 5 |
| 8. | I am comfortable in evaluating the quality of online medical research reports. | 1 | 2 | 3 | 4 | 5 |
| 9. | As a result of visiting health-related web sites, I am less anxious about my condition. | 1 | 2 | 3 | 4 | 5 |
| 10. | I trust online advice given by a Medical Doctor (MD). | 1 | 2 | 3 | 4 | 5 |
| 11. | I like to read online biographies of other patients that have had my condition. | 1 | 2 | 3 | 4 | 5 |
| 12. | I trust online healthcare advertising that has been sponsored by pharmaceutical companies. | 1 | 2 | 3 | 4 | 5 |
| 13. | As a result of visiting health-related web sites, I am less depressed about my condition. | 1 | 2 | 3 | 4 | 5 |
| 14. | I trust online advice given by a Registered Nurse (RN). | 1 | 2 | 3 | 4 | 5 |
| 15. | I will give my name to an online healthcare website if I will receive personalized information. | 1 | 2 | 3 | 4 | 5 |
| 16. | As a result of visiting health-related web sites, I have less uncertainty about my condition. | 1 | 2 | 3 | 4 | 5 |
| 17. | I trust online advice given by a Registered Dietitian (RD) | 1 | 2 | 3 | 4 | 5 |
| 18. | I like to exchange private e-mail with other patients who have my condition. | 1 | 2 | 3 | 4 | 5 |
| 19. | I trust a site more that discloses a healthcare professional’s financial interests in the site. | 1 | 2 | 3 | 4 | 5 |
| 20. | As a result of visiting health-related web sites, I feel less stress about my condition. | 1 | 2 | 3 | 4 | 5 |
| 21. | I trust online advice given by a Registered Pharmacist (RPh). | 1 | 2 | 3 | 4 | 5 |
| 22. | I am satisfied with the amount of support I receive online from other patients regarding my condition. | 1 | 2 | 3 | 4 | 5 |
| 23. | I tend to trust the products that other patients sell online. | 1 | 2 | 3 | 4 | 5 |
| 24. | As a result of visiting health-related web sites, my quality of life has improved. | 1 | 2 | 3 | 4 | 5 |
| 25.. | I feel better psychologically after meeting other patients online who have my condition. | 1 | 2 | 3 | 4 | 5 |
| 26. | I   tend to trust a site more that has a seal of approval, even if I don’t know the organization that is awarding it. | 1 | 2 | 3 | 4 | 5 |
| 27. | As a result of visiting health-related web sites, I am more accepting of my condition. | 1 | 2 | 3 | 4 | 5 |
| 28. | I like to attend online chats with health experts about my condition. | 1 | 2 | 3 | 4 | 5 |
| 29. | The more times I visit a web site, the more comfortable I am talking to others about my condition. | 1 | 2 | 3 | 4 | 5 |
| 30. | I trust a site that has been endorsed by a health authority. | 1 | 2 | 3 | 4 | 5 |
| 31. | As a result of visiting health-related web sites, I feel more hope about my condition. | 1 | 2 | 3 | 4 | 5 |
| 32 | I trust online summaries of health research articles even when I am not told who wrote them. | 1 | 2 | 3 | 4 | 5 |
| 33. | I would like to meet more patients online with my condition. | 1 | 2 | 3 | 4 | 5 |
| 34. | I am comfortable in evaluating health professional’s credentials. | 1 | 2 | 3 | 4 | 5 |
| 35. | As a result of visiting health-related web sites, I am better able to cope with my condition. | 1 | 2 | 3 | 4 | 5 |
| 36. | I feel that online health information is at a comfortable comprehension level. | 1 | 2 | 3 | 4 | 5 |
| 37. | I like to give online support to other patients who have my condition. | 1 | 2 | 3 | 4 | 5 |
| 38. | I want to know how my online health information is going to be used before I will provide information. | 1 | 2 | 3 | 4 | 5 |
| 39. | I would like to talk to other patients with my condition in my local area. | 1 | 2 | 3 | 4 | 5 |
| 40. | I tend to trust a site more that has been endorsed by a national association, even if I don’t know anything about the association. | 1 | 2 | 3 | 4 | 5 |
| 41. | I trust online reports of medical studies that have already been published in a journal. | 1 | 2 | 3 | 4 | 5 |
| 42 | I feel less alone after I meet other patients with my condition. | 1 | 2 | 3 | 4 | 5 |
